# Supplementary material for: Population Pharmacokinetic Study of Benzylpenicillin in Critically Unwell Adults
Source: Antibiotics (Basel). 2023 Mar 24;12(4):643. doi: 10.3390/antibiotics12040643 (PMC10135101; doi:10.3390/antibiotics12040643)
Supplement: Supplementary file 1 [file antibiotics-12-00643-s001.zip › antibiotics-2252113-supplementary.pdf]

Supplementary Material

Table S1: Summary of covariate model building

Run No.	Compartments	Model	OFV	Δ OFV	Comments
1	1	Basic model	296.4	-	
**2**	**2**	**Basic model**	**192.0**	**-**	**Accepted**
3	3	Basic model	189.4	-	V1 0.25 L70 kg; Q1 27300 L/h/70 kg: suggesting insignificant compartment
**4**	**2**	**Model 2 + Creatinine on CL**	**172.9**	**-19.1**	**Accepted**
5	2	Model 4 + BMI on V1	172.1	-0.8	Rejected
6	2	Model 4 + Height on V1	172.9	0	Rejected
7	2	Model 4 + Height on CL	168.9	-4.0	Large increase in uncertainty, therefore rejected (RSE on height covariate effect is 5119%)
8	2	Model 4 + Height on V2	172.7	-0.2	Rejected
9	2	Model 4 + Albumin on V1	172.3	-0.6	Rejected
10	2	Model 4 + Albumin on Q	171.3	-1.6	Rejected
11	2	Model 4+ Albumin on V2	170.6	-2.3	Rejected
12	2	Model 4 + Temperature on V1	170.0	-2.9	Rejected
13	2	Model 4 + Temperature on V2	162.7	-10.2	Large increase in uncertainty on other parameters, therefore rejected
14	2	Model 4 + Temperature on Q	171.0	-1.9	Rejected
15	2	Model 4 + APACHE on V1	172.1	-0.8	Rejected
16	2	Model 4 + APACHE on V2	172.7	-0.2	Rejected
17	2	Model 4 + SEX on CL	172.6	-0.3	Rejected
18	2	Model 4 + SEX on CL	172.9	0	Rejected
19	2	SEX on V1	172.9	0	Rejected

Table S2: Median %ft > MIC for various MICs with different dosing regimens

MIC (mg/L)	1200 mg bolus 4-hourly	2400 mg bolus 4-hourly	1200 mg bolus followed by 6000 mg infusion/24 hours	7200 mg infusion/24 hours	1200 mg infusion over 2-hours, 4-hourly
**0.125**	1.00	1.00	1.00	1.00	1.00
**0.25**	1.00	1.00	1.00	1.00	1.00
**0.5**	0.99	1.00	1.00	1.00	1.00
**1.0**	0.74	0.99	1.00	0.99	0.99
**2.0**	0.49	0.74	1.00	0.98	0.76
**4.0**	0.30	0.49	0.20	0.90	0.54
**8.0**	0.17	0.30	0.04	0.00	0.02
**16.0**	0.07	0.17	0.01	0.00	0.00
**32.0**	0.02	0.07	0.00	0.00	0.00
